# Increased Risk of Breakthrough SARS-CoV-2 Infections in Patients with Colorectal Cancer: A Population-Based Propensity-Matched Analysis

**DOI:** 10.3390/jcm13092495

**Published:** 2024-04-24

**Authors:** Saqr Alsakarneh, Fouad Jaber, Hana Qasim, Abdallah Massad, Hamza Alzghoul, Yazan Abboud, Dushyant Singh Dahiya, Mohammad Bilal, Aasma Shaukat

**Affiliations:** 1 Department of Medicine, University of Missouri, Kansas City, MO 64110, USA; fouad.jaber.md@gmail.com (F.J.); hqasim@umkc.edu (H.Q.); 2 Department of Medicine, University of Texas Medical Branch, Galveston, TX 77555, USA; abmassad@utmb.edu; 3 Department of Medicine, University of Central Florida, Orlando, FL 32816, USA; hamzehzghoolmd96@gmail.com; 4 Department of Medicine, Rutgers University School of Medicine, Newark, NJ 07103, USA; ya296@njms.rutgers.edu; 5 Division of Gastroenterology, Hepatology and Motility, University of Kansas, Lawrence, KS 66045, USA; ddahiya@kumc.edu; 6 Division of Gastroenterology, Hepatology, and Nutrition, University of Minnesota Medical Center, Minneapolis, MN 55455, USA; billa17@hotmail.com; 7 Division of Gastroenterology, Department of Medicine and Population Health, NYU Grossman School of Medicine, New York, NY 10016, USA; aasma.shaukat@nyulangone.org

**Keywords:** colorectal cancer, CRC, breakthrough, SARS-CoV-2, COVID-19

## Abstract

**Background/Objectives**: This study aimed to investigate the association between colorectal cancer (CRC) and the risk of breakthrough respiratory syndrome coronavirus 2 (SARS-CoV-2) infection in vaccinated patients with CRC. **Methods**: This retrospective cohort study used the TriNetX research network to identify vaccinated patients with CRC. Patients were matched using propensity score matching (PSM) and divided into patients with CRC and control (without history of CRC) groups. The primary outcome was the risk of breakthrough SARS-CoV-2 in vaccinated patients. The secondary outcome was a composite of all-cause emergency department (ED) visits, hospitalization, and death during the follow-up period after the diagnosis of COVID-19. **Results**: A total of 15,416 vaccinated patients with CRC were identified and propensity matched with 15,416 vaccinated patients without CRC. Patients with CRC had a significantly increased risk for breakthrough infections compared to patients without CRC (aOR = 1.78; [95% CI: 1.47–2.15]). Patients with CRC were at increased risk of breakthrough SARS-CoV-2 infections after two doses (aOR = 1.71; [95% CI: 1.42–2.06]) and three doses (aOR = 1.36; [95% CI: 1.09–1.69]) of SARS-CoV-2 vaccine. Vaccinated patients with CRC were at a lower risk of COVID-19 infection than unvaccinated CRC patients (aOR = 0.342; [95% CI: 0.289–0.404]). The overall composite outcome (all-cause ED visits, all-cause hospitalization, and all-cause death) was 51.6% for breakthrough infections, which was greater than 44.3% for propensity score-matched patients without CRC (aOR = 1.79; [95% CI: 1.29–2.47]). **Conclusions**: This cohort study showed significantly increased risks for breakthrough SARS-CoV-2 infection in vaccinated patients with CRC. Breakthrough SARS-CoV-2 infections in patients with CRC were associated with significant and substantial risks for hospitalizations.

## 1. Introduction

In the initial phases of the pandemic, data suggested an increased susceptibility of cancer patients to severe SARS-CoV-2 infection [1,2]. Ongoing vaccination efforts against SARS-CoV-2 have played a crucial role in mitigating morbidity and mortality rates, with a specific emphasis on inoculating patients with cancer to avoid severe outcomes in this population. In general, cancer patients are more susceptible to infections due to disrupted normal anatomical barriers and compromised cellular and humoral immunity, which are attributable to both the cancer itself and its treatment. While initial data on seroconversion and titers of anti-mRNA antibodies post-SARS-CoV-2 vaccination are promising for solid malignancies [3,4], concerns persist regarding the durability of this immune response.

Despite ongoing vaccination efforts, numerous studies have revealed waning immunity and an increased incidence of breakthrough SARS-CoV-2 infections in patients with cancer [5,6]. The efficacy of vaccines in patients with cancer is influenced by various factors intrinsic to the disease. Patients with cancer often exhibit immune dysfunction due to tumor-induced alterations in immune cell populations and cytokine profiles [7]. This compromised immunity extends to vaccine responses, potentially resulting in attenuated immunity following vaccination [8]. Moreover, the impact of cancer treatments such as chemotherapy further complicates vaccine efficacy by suppressing immune function and altering the timing of vaccination relative to treatment cycles [9]. Monitoring vaccine responses in cancer patients is challenging due to underlying comorbidities and the dynamic nature of immune function within the tumor microenvironment. Additionally, the potential for cancer cells to harbor molecular alterations that confer resistance to vaccination emphasizes the need for tailored immunization strategies in this population [10].

With the emergence of new variants, there has been a focus on assessing the risks and identifying vulnerable populations to breakthrough SARS-CoV-2 infection [11]. Previous data indicated that SARS-CoV-2 infection contributes significantly to morbidity and mortality in patients with colorectal cancer (CRC) [12]. Patients with cancer, particularly those undergoing active treatment or with advanced disease, may face an increased risk of severe SARS-CoV-2 outcomes due to compromised immune function [13]. In addition to vaccination, emerging pharmacological treatments such as monoclonal antibodies, including tixagevimab/cilgavimab, hold promise for preventing disease progression and reducing the severity of SARS-CoV-2 in eligible patients [14,15]. These therapies target specific viral components and have demonstrated efficacy in reducing viral load and hospitalization rates in high-risk individuals, including those with cancer [16].

We hypothesize that patients with CRC are at a higher risk of breakthrough SARS-CoV-2 infection and severe outcomes. Using large, nationally representative data from the United States (US), we aim to address these knowledge gaps by evaluating the relationship between CRC and vaccination efficacy.

## 2. Materials and Methods

### 2.1. Database Description

We conducted a retrospective cohort study utilizing the TriNetX (Cambridge, MA, USA) Analytics Network Platform. TriNetX is a multi-institutional global federated research network that contains de-identified data from more than 105 million patients within 61 health care organizations (HCOs) across the US. [17]. The TriNetX web portal can be used to perform cohort selection, propensity score matching, and time trend analysis to compare outcomes between cohorts. The de-identification process is determined and performed at the network level by a qualified expert as defined in the Health Insurance Portability and Accountability Act Privacy Rule (HIPAA). Rigorous quality assurance is achieved at the time of extraction of data from electronic health records (EHRs), in a systemic and standardized format, before inclusion in the database. Based on the recommendations of the National Human Research Protections Advisory Committee, this study was exempted from IRB approval because it involved publicly available de-identified data [18]. TriNetX has been previously used and validated for studying SARS-CoV-2 infections in the US [19,20]. This study followed the Strengthening the Reporting of Observational Studies in Epidemiology (STORBE) reporting guidelines [21].

### 2.2. Study Participants and Cohorts

A real-time search and analysis of the US Collaborative Network in the TriNetX platform were conducted through February 1, 2024. The CRC cohort included patients who fulfilled the following inclusion criteria: (a) were aged 18 years and older; (b) had an International Classification of Disease, Ninth Revision and Tenth Revision, Clinical Modification (ICD-10-CM) code in their EHR for CRC (Supplementary Table S1); (c) had documented evidence of vaccination in their EHRs (received two doses of the COVID-19 messenger RNA vaccine or a single dose of the COVID-19 viral vector vaccine); and (d) did not contract COVID-19 infection prior to vaccination. This case definition was used and validated in previously published studies [5,20].

**Figure 1:**
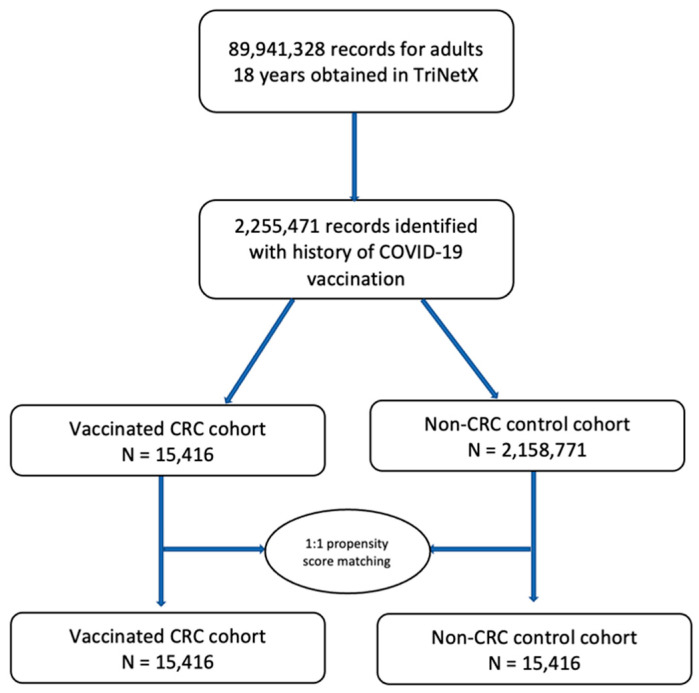
Flowchart for cohort identification of patients in the colorectal cancer (CRC) cohort and non-CRC control cohort.

The control cohort was stratified into two dichotomous cohorts: (1) cohort 1, in which patients had no medical encounters for CRC but were vaccinated against COVID-19; and (2) cohort 2, in which patients had medical encounters for CRC and did not receive the COVID-19 vaccine. The three cohorts were propensity score matched for covariates (Figure 1).

### 2.3. Covariates

The covariates used in this study and their standardized names, codes, and data types are described in Supplementary Table S1. The CRC and control groups were created using the following 1:1 propensity score matching variables: (a) demographic information (age, sex, race, and ethnicity) [22,23,24]; (b) social determinants of adverse health outcomes (SDHOs); (c) behavioral factors (tobacco smoking, alcohol abuse); (d) vaccine types; (e) cancer treatment types; and (f) comorbidities that are related to SARS-CoV-2 morbidity and mortality [25].

### 2.4. Study Aims and Outcomes

The primary aim of the study was to compare the risk of SARS-CoV-2 breakthrough infections after vaccination between the CRC cohort and the control cohort. Patients were considered to have breakthrough SARS-CoV-2 infection if they were diagnosed with SARS-CoV-2 infection 14 days to 6 months after vaccination [26]. The status of breakthrough SARS-CoV-2 infection was based on the laboratory test-confirmed presence of “SARS coronavirus 2 and related RNA” (TNX:LAB:9088) or the International Classification of Disease-10 Genomes (ICD-10) diagnosis code “COVID-19” (U07.1). The TriNetX database allows temporal associations between different groups within a cohort to be created. This functionality allowed us to identify patients who had SARS-CoV-2 infection after receiving the SARS-CoV-2 vaccine.

The secondary outcomes included (1) a composite of any emergency room (ER) visits, hospitalization, or death [27] and (2) ER visits, all-cause hospitalization, all-cause death, all-cause critical care admission, and all-cause intubation, individually. These outcomes were observed during the follow-up period, spanning 30 days after the breakthrough SARS-CoV-2 infection [27]. This case definition was previously used and validated with 98% sensitivity and 99% specificity in the US hospital setting [28]. In a separate subgroup analysis, we evaluated the risk of SARS-CoV-2 infection and severe outcomes between vaccinated and unvaccinated patients with CRC.

### 2.5. Statistical Analyses

All the statistical tests were performed using the built-in functions of the TriNetX Advanced Analytics Platform. The characteristics of the two groups are presented as the mean ± standard deviation (SD) or frequency and proportion. One-to-one (1:1) propensity score matching was performed to control for the covariate between the two groups. The TriNetX platform utilizes input matrices of user-identified covariates to conduct logistic regression analysis in order to obtain propensity scores for all individual subjects. The propensity scores generated were used to match patients using greedy nearest-neighbor algorithms with a caliper width of 0.1 for the pooled standard deviations. TriNetX randomizes the order of rows to eliminate bias resulting from nearest-neighbor algorithms. After propensity score matching, the risk of each outcome was calculated and is expressed as an adjusted odds ratio (aOR) with a 95% confidence interval (CI). Two-sided *p*-values < 0.05 were considered statistically significant.

## 3. Results

### 3.1. Patient Characteristics

A total of 15,416 vaccinated CRC patients were identified (mean age = 68.3 ± 12.7 years, female 47.4%) and matched with 15,416 patients without CRC. Among the CRC-vaccinated patients, 69.7% were White individuals, 11.6% were Black individuals, and 7.9% were Hispanic/Latino individuals. In this cohort, 11,168 patients received two doses, and 2311 patients received three doses of the COVID-19 mRNA vaccine. A full description of the demographic and comorbidity data before and after propensity score matching is shown in Table 1.

### 3.2. Comparison of Breakthrough SARS-CoV-2 Infections between Patients with and without Colorectal Cancer

**Figure 2:**
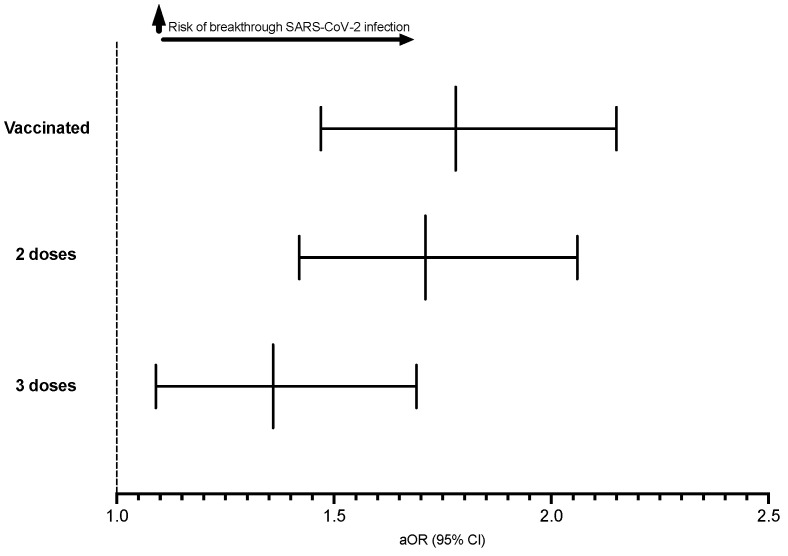
Risk of COVID-19 breakthrough infection in patients with CRC compared to patients without CRC.

Overall, vaccinated CRC patients were significantly more prone to breakthrough infections than vaccinated patients without CRC after matching for demographic variables, SDHOs, comorbidities, and vaccine and cancer treatment types (aOR = 1.78; [95% CI: 1.47–2.15]). Subgroup analysis based on the number of vaccines yielded similar findings. Patients were at increased risk of breakthrough SARS-CoV-2 infections after two doses (aOR = 1.71; [95% CI: 1.42–2.06]) and three doses (aOR = 1.36; [95% CI: 1.09–1.69]) (Figure 2).

### 3.3. Outcomes of Breakthrough SARS-CoV-2 Infections

Among patients with CRC, the overall composite outcome (all-cause ED visits, all-cause hospitalization, and all-cause death) was 51.6% for breakthrough infections, which was greater than 44.3% for propensity score-matched patients without CRC (aOR = 1.79; [95% CI: 1.29–2.47]). Furthermore, patients with CRC had a significantly greater incidence of composite outcomes than did those in the control group (log rank test, *p* < 0.0001). Although mortality and ICU admissions were not different between the two groups, the CRC patients had a significantly greater risk of hospitalization (aOR = 3.01; [95% CI = 2.1–4.3]) (Table 2).

According to our subgroup analysis, the composite outcome was greater in CRC patients than in patients without CRC after vaccination with two doses (aOR = 1.84; [95% CI = 1.29–2.62]) or three doses (aOR = 1.77; [95% CI = 1.09–2.88]). Patients who were vaccinated for three doses were at higher risk of 30-day all-cause hospitalization if they developed breakthrough infections (aOR = 3.07; [95% CI: 1.78–5.28]). No significant differences were observed in a subgroup analysis based on sex (men vs. women) (Table 2). Supplemental Table S2 shows the clinical and demographic characteristics of the patients included in the evaluation of these outcomes.

### 3.4. Vaccinated vs. Unvaccinated Patients with CRC

Vaccinated patients with CRC were at a lower risk of COVID-19 infection than unvaccinated CRC patients (aOR = 0.342; [95% CI: 0.289–0.404]). On a subgroup analysis, the composite outcome was lower in vaccinated patients (aOR = 0.724; [95% CI: 0.535–0.98]) (Table 3).

## 4. Discussion

Our analysis, using real-world evidence data, demonstrated a greater risk of breakthrough COVID-19 infections in vaccinated patients with CRC than in vaccinated patients without a history of CRC. Overall, rates of breakthrough COVID-19 infections were greater in CRC patients regardless of the number of vaccine doses, indicating that vaccines are less effective in CRC patients. This trend may reflect waning immunity of vaccines in the CRC population [29,30]. Our study also showed higher all-cause hospitalization rates among CRC patients with breakthrough COVID-19 infection than among those without a history of CRC.

In our analysis, to evaluate whether CRC itself increases the risk for breakthrough COVID-19 infections and worse outcomes, we conducted propensity score matching for various covariates that were linked to an increased risk of COVID-19 infections. Interestingly, as immunosuppressive treatments and comorbidities were controlled by using propensity score matching, CRC patients were still at increased risk of breakthrough COVID-19 infections, suggesting that CRC itself might play a role in the increased risk of infections, irrespective of cancer treatment and comorbidities.

While cancer patients are known to be at increased susceptibility to infections in general [31], the reasons behind the increase in the incidence of breakthrough COVID-19 infections in vaccinated CRC patients remain unclear. The increased risk of breakthrough infections in CRC patients following vaccination may be attributed to several biological mechanisms. Potential etiologies include the immunosuppressive effect of cancer treatment combined with chemotherapy and immunotherapy [32,33], which could affect seroconversion and antibody titers in cancer patients receiving active treatment. The immunosuppressive effects of CRC itself, compounded by the tumor microenvironment’s inhibitory signals, may impair vaccine-induced immune responses. Studies have shown that CRC patients often exhibit dysfunctional immune cell populations and altered cytokine profiles, which can compromise the generation of robust and durable immunity following vaccination [7,34]. Furthermore, chemotherapy, radiation therapy, and surgical interventions can all exert immunosuppressive effects, disrupting the balance of immune homeostasis and impairing vaccine responsiveness [35]. Chemotherapeutic agents, in particular, may induce cytotoxic effects on immune cells and hinder antigen presentation, thereby diminishing the efficacy of vaccination [35,36].

The impaired cellular immunity in cancer patients, regardless of treatment status [33], contributes to the shortened durability of their immunity after receiving the COVID-19 vaccine and accordingly increases the need for booster doses to maintain their antibody titer. Furthermore, the emergence of multiple variants of COVID-19 infections over time, against which vaccines might not be as effective, could contribute to this trend.

Since some of the most significant outcomes of COVID-19 infection include emergency department (ED) visits, hospitalizations, and mortality, we integrated these outcomes into a composite measure and evaluated it as a secondary outcome in our study [27]. Our study revealed an increased risk of the composite outcome among CRC patients. This trend was consistent in the two-dose group and the three-dose group. Moreover, CRC patients had higher rates of all-cause hospitalization, regardless of the number of vaccine doses administered. These trends result in an increased burden on healthcare resource utilization and higher morbidity in this patient population. These findings indicate that CRC may be associated with a greater risk of post-acute breakthrough SARS-CoV-2 infection. Our study showed no increase in the risk of mortality or ICU admission between the CRC patients and non-CRC patients.

Our study has several limitations. First, it is imperative to acknowledge the observational and retrospective design inherent in this study, which could introduce biases associated with case selection, reporting, testing, and follow-up. However, using data exclusively from one database, TriNetX, to compare different cohorts, these issues are anticipated to exert minimal impact on the integrity of the relative risk analyses conducted. Second, we could not evaluate the immune response to the COVID-19 vaccine among cancer patients, such as by quantifying antibody titers, which limits our ability to determine the underlying reasons for breakthrough infections and to determine the necessity for booster doses accordingly. Additionally, using data from the TriNetX network, primarily from US health care organizations, may limit the applicability of findings to other health care systems or countries. The strengths of our study include the use of large, propensity-matched, US-based multi-institutional cohorts.

## 5. Conclusions

In conclusion, our retrospective cohort study provides real-world evidence that vaccinated colorectal cancer patients are at a significantly higher risk for breakthrough COVID-19 infections and present higher rates of adverse clinical outcomes such as hospitalization. These findings emphasize the importance of continuing efforts and exploring tailored strategies for cancer patients, especially those with colorectal cancer, against the ongoing threat of breakthrough COVID-19 infections. As we navigate the complexities of vaccination and infectious disease management, our study contributes valuable insights that may inform public health measures and clinical interventions for this vulnerable patient population. Future studies are warranted to evaluate the biological mechanisms underlying breakthrough infections among colorectal cancer patients and to assess interventions aimed at mitigating the increased risk observed in CRC patients.

## Figures and Tables

**Table 1:** Baseline characteristics of patients in the CRC cohort and non-CRC control cohort before and after propensity score matching.

	Before Matching			After Matching		
	With CRC (n, %) n = 15,416	Without CRC (n, %) n = 2,158,771	*p* Value	With CRC (n, %) n = 15,416	Without CRC (n, %) n = 15,416	*p* Value
**Demographics**						
Age (mean)	69.6 ± 12.5	53.1 ± 19.5	<0.001	69.6 ± 12.5	70.2 ± 12.2	<0.001
Female	7492 (48.60%)	1,128,860 (52.30%)	<0.001	7491 (48.60%)	7695 (49.90%)	0.02
Hispanic or Latino	1219 (7.90%)	329,008 (15.20%)	<0.001	1219 (7.90%)	1106 (7.20%)	0.015
White	10,743 (69.70%)	1,386,991 (64.20%)	<0.001	10,742 (69.70%)	10,995 (71.30%)	0.002
Black or African American	1793 (11.60%)	216,367 (10.00%)	<0.001	1793 (11.60%)	1808 (11.70%)	0.79
Asian	1062 (6.90%)	167,199 (7.70%)	<0.001	1062 (6.90%)	993 (6.40%)	0.115
**Diagnosis**						
Obesity	4301 (27.90%)	278,403 (12.90%)	<0.001	4300 (27.90%)	4469 (29.00%)	0.033
Diabetes mellitus	4262 (27.60%)	224,236 (10.40%)	<0.001	4261 (27.60%)	4577 (29.70%)	<0.001
Hypertension	9962 (64.60%)	563,266 (26.10%)	<0.001	9961 (64.60%)	10,563 (68.50%)	<0.001
Hyperlipidemia	7356 (47.70%)	390,934 (18.10%)	<0.001	7355 (47.70%)	7815 (50.70%)	<0.001
Heart failure	1959 (12.70%)	71,271 (3.30%)	<0.001	1959 (12.70%)	2012 (13.10%)	0.368
CLRD	3839 (24.90%)	234,019 (10.80%)	<0.001	3839 (24.90%)	3969 (25.70%)	0.089
IHD	4137 (26.80%)	169,624 (7.90%)	<0.001	4137 (26.80%)	4147 (26.90%)	0.898
CKD	2889 (18.70%)	105,588 (4.90%)	<0.001	2888 (18.70%)	2982 (19.30%)	0.173
Diseases of liver	3447 (22.40%)	96,400 (4.50%)	<0.001	3446 (22.40%)	3348 (21.70%)	0.178
CVD	1880 (12.20%)	87,249 (4.00%)	<0.001	1880 (12.20%)	2009 (13.00%)	0.027
SDHOs	605 (3.90%)	34,431 (1.60%)	<0.001	605 (3.90%)	565 (3.70%)	0.233
HIV	189 (1.20%)	9177 (0.40%)	<0.001	189 (1.20%)	180 (1.20%)	0.637
Dementia	388 (2.50%)	14,252 (0.70%)	<0.001	388 (2.50%)	381 (2.50%)	0.798
Substance use disorders	2634 (17.10%)	168,404 (7.80%)	<0.001	2633 (17.10%)	2769 (18.00%)	0.042
Depression	2983 (19.40%)	199,924 (9.30%)	<0.001	2982 (19.30%)	3155 (20.50%)	0.014
Anxiety	4038 (26.20%)	298,874 (13.80%)	<0.001	4037 (26.20%)	4294 (27.90%)	0.001
Alcohol abuse	465 (3.00%)	28,708 (1.30%)	<0.001	465 (3.00%)	463 (3.00%)	0.947
Tobacco use	737 (4.80%)	45,197 (2.10%)	<0.001	737 (4.80%)	736 (4.80%)	0.979
**Procedure**						
SCT	57 (0.40%)	2496 (0.10%)	<0.001	57 (0.40%)	68 (0.40%)	0.324
Chemotherapy	4959 (32.20%)	109,228 (5.10%)	<0.001	4958 (32.20%)	4592 (29.80%)	<0.001
Targeted Therapy	1837 (11.90%)	65,534 (3.00%)	<0.001	1837 (11.90%)	1801 (11.70%)	0.525
Radiation	1419 (9.20%)	21,552 (1.00%)	<0.001	1418 (9.20%)	1146 (7.40%)	<0.001
Hormone Therapy	1011 (6.60%)	58,354 (2.70%)	<0.001	1011 (6.60%)	1016 (6.60%)	0.909
CAR-T therapy	10 (0.10%)	49 (0.00%)	<0.001	10 (0.10%)	10 (0.10%)	1
**Medication**						
pembrolizumab	150 (1.00%)	1444 (0.10%)	<0.001	150 (1.00%)	125 (0.80%)	0.13
nivolumab	76 (0.50%)	993 (0.00%)	<0.001	76 (0.50%)	59 (0.40%)	0.143
cemiplimab	10 (0.10%)	41 (0.00%)	<0.001	10 (0.10%)	10 (0.10%)	1
atezolizumab	11 (0.10%)	291 (0.00%)	<0.001	11 (0.10%)	12 (0.10%)	0.835
avelumab	10 (0.10%)	21 (0.00%)	<0.001	10 (0.10%)	10 (0.10%)	1
durvalumab	24 (0.20%)	261 (0.00%)	<0.001	24 (0.20%)	20 (0.10%)	0.546
ipilimumab	31 (0.20%)	426 (0.00%)	<0.001	31 (0.20%)	28 (0.20%)	0.696

Abbreviations: CRC: colorectal cancer; CLRD: chronic lower respiratory diseases; IHD: ischemic heart diseases; CKD: chronic kidney diseases; HIV: human immunodeficiency virus; CVD: cerebrovascular diseases; SDHOs: social determinants of adverse health outcomes; SCT: stem cell transplant; CAR-T: chimeric antigen receptor T-cell therapy.

**Table 2:** Outcomes of breakthrough COVID-19 infections in patients with CRC compared to patients without CRC after 1:1 propensity score matching expressed as adjusted odds ratio (aOR) with 95% confidence intervals (CI).

Outcome	aOR	95% CI	*p* Value
Composite outcome	1.79	(1.298, 2.469)	0.0004
Mortality	1	0.411, 2.435)	1
Hospitalization	3.01	(2.101, 4.318)	<0.0001
Emergency department	0.763	(0.518, 1.123)	0.1690
ICU	1	(0.411, 2.435)	1
Intubation	1	(0.411, 2.435)	1
**2 Doses**			
Composite outcome	1.84	(1.29, 2.624)	0.0007
Mortality	1	(0.41, 2.44)	1
Hospitalization	3.261	(2.172, 4.896)	<0.0001
ED	0.847	(0.553, 1.299)	0.4466
ICU	1	(0.41, 2.44)	1
Intubation	1	(0.41, 2.44)	1
**3 Doses**			
Composite outcome	1.77	(1.089, 2.875)	0.0205
Mortality	1	(0.41, 2.44)	1
Hospitalization	3.065	(1.781, 5.277)	<0.0001
ED	0.558	(0.282, 1.104)	0.0907
ICU	1	(0.41, 2.44)	1
Intubation	1	(0.41, 2.44)	1
**Age**	**<65 vs. ≥65**		
Composite outcome	1.058	(0.548, 2.043)	0.8667
Mortality	1	(0.39, 2.566)	1
Hospitalization	0.829	(0.415, 1.659)	0.5963
ED	1.457	(0.678, 3.129)	0.3334
ICU	1	(0.39, 2.566)	1
Intubation	1	(0.39, 2.566)	1
**Gender**	**Male vs. Female**		
Composite outcome	1.583	(0.397, 2.522)	0.1129
Mortality	1	(0.397, 2.522)	1
Hospitalization	1.724	(0.951, 3.127)	0.717
ED	2.05	(1.026, 4.096)	0.0399
ICU	1	(0.397, 2.522)	1
Intubation	1	(0.397, 2.522)	1

**Table 3:** Risk of breakthrough infection and severe outcomes between vaccinated and unvaccinated patients with CRC after 1:1 propensity score matching expressed as adjusted odds ratio (aOR) with 95% confidence intervals (CI).

Vaccinated vs. Unvaccinated	aOR	95% CI	*p* Value
Breakthrough COVID-19 infection	0.342	(0.289, 0.404)	<0.0001
Composite outcome	0.724	(0.535, 0.981)	0.0371
Mortality	1.477	(0.675, 3.232)	0.3261
Hospitalization	0.884	(0.64, 1.223)	0.4573
Emergency department	0.929	(0.664, 1.3)	0.6683
ICU	0.77	(0.408, 1.455)	0.4204
Intubation	1.15	(0.552, 2.395)	0.7088

## Data Availability

Data are available upon reasonable request from the corresponding author.

## Supplementary Materials

The following supporting information can be downloaded at https://www.mdpi.com/article/10.3390/jcm13092495/s1: Table S1: Clinical diagnosis other codes used to determine the status of variables in the TriNetX database; Table S2: Clinical and demographic characteristics of patients with breakthrough SARS-CoV-2 infections.
