# Supplementary material for: Increased Risk of Breakthrough SARS-CoV-2 Infections in Patients with Colorectal Cancer: A Population-Based Propensity-Matched Analysis
Source: J Clin Med. 2024 Apr 24;13(9):2495. doi: 10.3390/jcm13092495 (PMC11084503; doi:10.3390/jcm13092495)
Supplement: Supplementary file 1 [file jcm-13-02495-s001.zip › jcm-2947094-supplementary.pdf]

Supplementary Table S1: Clinical diagnosis other codes used to determine the status of variables in the TriNetX database.

Covariate	Name, code
Age at Index	Age at Index
Male	M
Female	F
White	White (Demographics: 2106-3)
Black or African American	Black or African American (Demographics: 2054-5)
Asian	Asian (Demographics: 2028-9)
Hispanic/Latino	Hispanic or Latino (Demographics: 2135-2)
Not Hispanic or Latino	Not Hispanic or Latino (Demographics: 2186-5)
Hypertension	Hypertensive diseases (ICD-10 code: I10-I16)
Heart diseases	Ischemic heart diseases (ICD-10 code: I20-I25)
Cerebrovascular diseases	Cerebrovascular diseases (ICD-10 code: I60-I69)
Type 2 diabetes	Type 2 diabetes mellitus (ICD-10 code: E11)
Obesity/overweight	Overweight and obesity (ICD-10 code: E66)
Chronic kidney diseases	Chronic kidney disease (CKD) (ICD-10 code: N18)
Chronic lower respiratory diseases	Chronic lower respiratory diseases (ICD-10 code: J40-J47)
Liver diseases	Diseases of liver (ICD-10 code: K70-K77)
HIV infection	Human immunodeficiency virus [HIV] disease (ICD-10 code: B20)
Dementia	Vascular dementia (F01),<br>Dementia in other diseases classified elsewhere (F02)<br>Unspecified dementia (F03) Alzheimer's disease (G30)
Depression	Depressive episode (F32)
Anxiety	Anxiety, dissociative, stress-related, somatoform and other nonpsychotic mental disorders (ICD-10 code: F40-F48)
Substance use disorders	Mental and behavioral disorders due to psychoactive substance use (F10-F19)
Alcohol abuse	Alcohol abuse (F10.1)
Tobacco use	Tobacco use (ICD-10 code: Z72.0)
Adverse socioeconomic determinants of health	Persons with potential health hazards related to socioeconomic and psychosocial circumstances (ICD-10 code: Z55-Z65) Z55: Problems related to education and literacy Z56: Problems related to employment and unemployment Z57: Occupational exposure to risk factors (noise, radiation, dust, other air contaminants, toxic agents in agriculture, extreme temperature, etc). Z58: Problems related to physical environment Z59: Problems related to housing and economic circumstances Z60: Problems related to social environment Z62: Problems related

	to upbringing Z63: Other problems related to primary support groups Z64-Z65: Problems related to psychosocial circumstances
Chemotherapy	Chemotherapy (code 1002)
Stem cell transplant	Stem Cell Transplant (code 1005)
Targeted therapy	Targeted Therapy (code 1003)
Radiation	Radiation (code 1001)
Hormone therapy	Hormone Therapy (code 1004)
Immunotherapy	Pembrolizumab (RxNorm code: 1547545), Nivolumab (1597876), Cemiplimab (2058826), Atezolizumab (1792776), Avelumab (1875534), Durvalumab (1919503), Ipilimumab (1094833) Chimeric antigen receptor T-cell (CAR-T) therapy (CPT code 1035206)
Colorectal cancer	C18-C20
Vaccines	Pfizer-BioNTech vaccine : Immunization administration by intramuscular injection of severe acute respiratory syndrome coronavirus 2 (SARSCoV-2) (Coronavirus disease [COVID-19]) vaccine, mRNALNP, spike protein, preservative free, 100 mcg/0.5mL dosage; second dose (CPT code: 0012A)<br><br>Moderna vaccine: Immunization administration by intramuscular injection of severe acute respiratory syndrome coronavirus 2 (SARSCoV-2) (Coronavirus disease [COVID-19]) vaccine, mRNALNP, spike protein, preservative free, 30 mcg/0.3mL dosage, diluent reconstituted; second dose (CPT code: 0002A)<br><br>Johnson & Johnson vaccine: Immunization administration by intramuscular injection of severe acute respiratory syndrome coronavirus 2 (SARSCoV-2) (coronavirus disease [COVID-19]) vaccine, DNA, spike protein, adenovirus type 26 (Ad26) vector, preservative free, 5x10^10 viral particles/0.5mL dosage, single dose (CPT code: 0031A)

Supplementary Table S2: Clinical and demographic characteristics of patients with breakthrough SARS-CoV-2 infections.

	Before Matching			After Matching		
	With CRC (n,%) n=1,795	Without CRC (n,%) n=154,443	P value	With CRC (n,%) n=1,794	Without CRC (n, %) n=1,794	P value
**Demographics**						
Age at Index	Mean: 67.8 +/- 12.9	Mean:55.5 +/- 19.3	<0.001	Mean: 67.8 +/- 12.9	Mean: 68.7 +/- 12.7	0.034
Female	881 (49.10%)	88,362 (57.20%)	<0.001	881 (49.10%)	857 (47.80%)	0.423
Hispanic or Latino	173 (9.60%)	18,715 (12.10%)	0.001	173 (9.60%)	151 (8.40%)	0.2
White	1,246 (69.40%)	103,778 (67.20%)	0.046	1,246 (69.50%)	1,237 (69.00%)	0.745
Black or African American	234 (13.00%)	22,821 (14.80%)	0.039	234 (13.00%)	242 (13.50%)	0.694
Asian	134 (7.50%)	8,518 (5.50%)	<0.001	133 (7.40%)	123 (6.90%)	0.517
**Diagnosis**						
Obesity	650 (36.20%)	47,256 (30.60%)	<0.001	650 (36.20%)	654 (36.50%)	0.89
Diabetes mellitus	591 (32.90%)	36,544 (23.70%)	<0.001	591 (32.90%)	612 (34.10%)	0.458
Essential hypertension	1,286 (71.60%)	78,490 (50.80%)	<0.001	1,285(71.60%)	1,308 (72.90%)	0.391
Hyperlipidemia	1,035 (57.70%)	62,036 (40.20%)	<0.001	1,035(57.70%)	1,061 (59.10%)	0.378
Heart failure	360 (20.10%)	19,306 (12.50%)	<0.001	360 (20.10%)	385 (21.50%)	0.303
Chronic lower respiratory diseases	614 (34.20%)	46,366 (30.00%)	<0.001	614 (34.20%)	635 (35.40%)	0.462
Ischemic heart diseases	701 (39.10%)	33,186 (21.50%)	<0.001	701 (39.10%)	733 (40.90%)	0.275
Chronic kidney disease (CKD)	492 (27.40%)	23,655 (15.30%)	<0.001	492 (27.40%)	541 (30.20%)	0.071
Diseases of liver	722 (40.20%)	22,526 (14.60%)	<0.001	721 (40.20%)	692 (38.60%)	0.322
Cerebrovascular diseases	322 (17.90%)	19,334 (12.50%)	<0.001	322 (17.90%)	345 (19.20%)	0.324
Adverse socioeconomic determinants of health	130 (7.20%)	9,073 (5.90%)	0.014	130 (7.20%)	122 (6.80%)	0.601
Human immunodeficiency virus [HIV] disease	22 (1.20%)	1,899 (1.20%)	0.988	22 (1.20%)	25 (1.40%)	0.66
Dementia	53 (3.00%)	3,285 (2.10%)	0.016	53 (3.00%)	63 (3.50%)	0.345
Substance use disorders	488 (27.20%)	32,561 (21.10%)	<0.001	488 (27.20%)	484 (27.00%)	0.881
Depression	507 (28.20%)	39,498 (25.60%)	0.01	507 (28.30%)	522 (29.10%)	0.58
Anxiety	673 (37.50%)	54,218 (35.10%)	0.035	673 (37.50%)	644 (35.90%)	0.315
Alcohol abuse	88 (4.90%)	7,041 (4.60%)	0.488	88 (4.90%)	96 (5.40%)	0.545
Tobacco use	112 (6.20%)	8,145 (5.30%)	0.069	112 (6.20%)	114 (6.40%)	0.891
**Procedure**						
Stem Cell Transplant	13 (0.70%)	872 (0.60%)	0.37	13 (0.70%)	14 (0.80%)	0.847

Chemotherapy	1,121 (62.50%)	24,402 (15.80%)	<0.001	1,120 (62.40%)	1,132 (63.10%)	0.679
Targeted Therapy	452 (25.20%)	14,647 (9.50%)	<0.001	452 (25.20%)	455 (25.40%)	0.908
Radiation	427 (23.80%)	6,285 (4.10%)	<0.001	426 (23.70%)	430 (24.00%)	0.875
Hormone Therapy	210 (11.70%)	12,974 (8.40%)	<0.001	210 (11.70%)	216 (12.00%)	0.757
Chimeric antigen receptor T-cell (CAR-T) therapy	0 (0.00%)	31 (0.00%)	0.548	0 (0.00%)	0 (0.00%)	--
**Medication**						
pembrolizumab	52 (2.90%)	596 (0.40%)	<0.001	52 (2.90%)	58 (3.20%)	0.561
nivolumab	19 (1.10%)	354 (0.20%)	<0.001	19 (1.10%)	21 (1.20%)	0.75
cemiplimab	10 (0.60%)	16 (0.00%)	<0.001	10 (0.60%)	0 (0.00%)	0.002
atezolizumab	10 (0.60%)	121 (0.10%)	<0.001	10 (0.60%)	10 (0.60%)	1
avelumab	0 (0.00%)	11 (0.00%)	0.721	0 (0.00%)	0 (0.00%)	--
durvalumab	10 (0.60%)	102 (0.10%)	<0.001	10 (0.60%)	10 (0.60%)	1
ipilimumab	10 (0.60%)	182 (0.10%)	<0.001	10 (0.60%)	10 (0.60%)	1
